# Novel Technologies Based on Supercritical Fluids for the Encapsulation of Food Grade Bioactive Compounds

**DOI:** 10.3390/foods9101395

**Published:** 2020-10-02

**Authors:** Stefan Klettenhammer, Giovanna Ferrentino, Ksenia Morozova, Matteo Scampicchio

**Affiliations:** Faculty of Science and Technology, Free University of Bolzano, Piazza Università 1, 39100 Bolzano, Italy; stefan.klettenhammer@unibz.it (S.K.); ksenia.morozova@unibz.it (K.M.); matteo.scampicchio@unibz.it (M.S.)

**Keywords:** novel technologies, supercritical fluids, encapsulation, food grade bioactive compounds

## Abstract

In recent years, the demand for nutritive, functional and healthy foods has increased. This trend has induced the food industry to investigate novel technologies able to produce ingredients with enhanced functional and physicochemical properties. Among these technologies, one of the most promising is the encapsulation based on supercritical fluids. Thanks to the inherent absence of organic solvent, the low temperature of the process to reach a supercritical state and the capacity to dissolve lipid soluble bioactives, the encapsulation with supercritical carbon dioxide represents a green technology to produce several functional ingredients, with enhanced stability, high load and tailored protection from environmental factors. Furthermore, from the fine-tuning of the process parameters like temperature, pressure and flow rate, the resulting functional ingredient can be easily designed to tailor the controlled release of the bioactive, or to reach specific levels of taste, odor and color. Accordingly, the aim of the present review is to summarize the state of the art of the techniques based on supercritical carbon dioxide for the encapsulation of bioactive compounds of food interest. Pros and cons of such techniques will be highlighted, giving emphasis to their innovative aspects that could be of interest to the food industry.

## 1. Introduction

Encapsulation is a common strategy to entrap active ingredients within a carrier material. In food formulation, it is very common to entrap sensitive actives like antioxidants, vitamins or unsaturated oils into a shell made from food grade polymers. The result is a powder, usually with enhanced storage stability and superior protection against light, temperature, pH or oxygen [1]. Recent developments on encapsulation technologies have contributed not only to enhance the chemical stability of the bioactive compounds, but also to tailor specific properties of the powder formulations, like their microstructures and the final rate of release [2].

The capacity to encapsulate active ingredients and protect them from degradation is of great economic importance. It is worth to note that functional ingredients reached a market of about 9.36 billion dollars in 2015 and the plan is to obtain about 41.74 billion dollars by 2021 with a computed annual growth rate of 6% [1]. The main food and dietary supplements are sold around the globe in the form of encapsulate comprise emulsions, dispersions and water-soluble powdered preparations. Nowadays, such encapsulated products can be found in the market as food ingredients and supplements [2]. However, some challenges are still open and mainly related to their efficiency to preserve the functional properties of the bioactive compounds during storage, processing, or even after the consumption and flow through the gastrointestinal tract.

From a technological point of view, the challenge to preserve the functional activity of the bioactive is even more complicated nowadays by the growing demand of powder ingredients that are free from solvents residues, show high flowability, little or no shrinkage in time, negligible diffusion of the active towards the surface and negligible impact on the food quality attributes once incorporated in the formulation.

Accordingly, the type of encapsulation technology plays a major role in the final success of a formulation. Through the years, several encapsulation technologies have been developed with the aim to protect bioactive compounds. The most relevant examples include spray drying, freeze drying, spray-chilling, extrusion, coacervation, electrospinning and fluidized bed. On the other hand, research studies on some other techniques, such as those using supercritical fluids, have shown only recently their performance and possible applicability at industrial scale [1,3,4].

Recent studies have revealed that supercritical fluids can be a further alternative technology for encapsulating active compounds [1]. Although most of the applications of these works have been limited so far to the production of pharmaceutical and cosmetic products, there is also a great potential to transfer such technology in food-related applications. In particular, encapsulation based on supercritical fluids offers the potential benefit to prepare powder formulations free from solvent traces, high encapsulation efficiencies, high active ingredient load and simple scale-up [1,5].

Therefore, the objective of this review is to give a detailed overview of supercritical fluid-based techniques for the production of encapsulated food-grade ingredients with enhanced functional properties, which have potential application in food products and developments at industrial scale for the food industry.

## 2. Techniques Used for the Encapsulation of Bioactive Compounds

Through the years, a number of technologies have been developed, like spray drying, spray-bed-drying, fluid-bed coating, spray-chilling, or spray-cooling, to encapsulate active agents. Most of them are based on a drying step as they involve a previous step of emulsification to solubilize the bioactive compounds in water or oil and produce water in oil emulsions, oil in water emulsions, or water in oil in water double emulsions [6].

Spray drying is the most widely used encapsulation technique in the food industry. It is a flexible, continuous and economical operation able to produce particles of good quality attributes with size less than 40 μm. However, this technique presents several disadvantages such as the complexity of the equipment, the non-uniform conditions achieved in the drying chamber and the not always easy control of the particle size of the particles [7]. About 80–90% of encapsulated products present on the market are produced by spray drying. The rest of them are mainly prepared by spray-chilling, vacuum, or freeze-drying just to name some.

In particular, vacuum and freeze-drying are processes often applied as alternative to spray drying. Vacuum drying is faster and cheaper compared to freeze drying as it operates at a temperature above the freezing point of the solvent. However, the produced particles are not uniform in shape and size. On the other hand, freeze-drying presents several disadvantages linked to the high energy input and long processing times required to obtain encapsulates. Moreover, during the process, a barrier with an open porous structure between the bioactive compound and its surroundings is often formed. This favors the formation of a high-porous wall, which offers poor protection when a prolonged release of the bioactive compound is required [8].

## 3. Bioactive Compounds Worth to Encapsulate

Bioactive compounds are usually extracted or recovered from plant or animal sources. Table 1 shows some of the most studied, including antioxidants, vitamins, pigments and essential or vegetable oils. They are usually added to foods to enrich their functional properties. However, their stability is generally low. Usually, processing conditions and long storage time are two of the most common factors responsible for the reduction of their functionality. The exposure to external factors, like temperature, light, oxygen and pH, causes the loss of bioactive functionality. In addition, there is a growing amount of evidence that shows how many functional compounds can greatly lose their bioavailability after consumption during their flow through the gastrointestinal tract [9,10].

As an example, polyphenols are highly affected by the alkaline conditions of the small intestine. Gayoso et al. [11] studied the bioaccessibility and antioxidant activity of rutin, caffeic acid and rosmarinic acid using filtration, centrifugation and dialysis as three different in vitro gastrointestinal digestion models. They observed a significant degradation of all compounds at the intestinal level [11]. Similarly, a pancreatic digestion was carried out on polyphenols from chokeberry juice. All the polyphenols were significantly altered during the pancreatic action [12].

### 3.1. Antioxidants and Vitamins

From the economical and nutritional point of view, two of the most important classes of food active ingredients include antioxidants and vitamins. Among the natural antioxidants, vitamins E, C and A, carotenoids and flavonoids are likely the most widely used [13].

When those molecules are used as food additives, they can control the rancidity development, maintain nutritional quality, retard the formation of toxic oxidation products and extend the shelf-life of foods [14]. Antioxidants and vitamins are known for their beneficial health effects. Several studies have tentatively attributed to many phenolic compounds, at different extents, some antioxidant, anticarcinogenic, anti-inflammatory, antimutagenic and antimicrobial activities [15].

However, it has been observed that such compounds lose their bioactivity and bioavailability when added to foods [16]. As an example, carotenoids are susceptible to oxidation induced by several agents such as light, heat, presence of metals or acids and so on. Consequently, the oxidative damage occurred to carotenoids, when added to functional foods, can be reflected as a loss of product quality and bioactivity [17,18].

Valerio et al. [19] studied the degradation of carotenoids in edible oils exposed at different temperatures from 110 to 150 °C along the heating time. A first order kinetic of degradation was detected for carotenoids, which was highly correlated to the high temperatures applied during the heating [19]. Similarly, a recent study showed that some polyphenols with antioxidant properties contained in the barley flour were degraded when used to produce baked fortified crackers. Indeed, the results reported a decrease of the concentration of some antioxidants such as procyanidin C, α-tocotrienols and ferulic acid in the final product due to the baking process [20].

### 3.2. Vegetable and Essential Oils

Vegetable oils are edible mixtures of triglycerides, generally liquids at room temperature and typically extracted from seeds. They are rich in polyunsaturated fatty acids, which provide them some nutritional health claims [21,22]. For these reasons, they are often used as food ingredients. However, a major challenge in their use is their susceptibility toward oxidative deterioration. During oxidation, the oil leads to the production of peroxides, which are responsible for the evolution of unpleasant odors. This, in turn, leads to negative effects on the sensory properties, shelf life, and consistency of foods [1,3].

As an example, crude soybean oil showed a longer oxidative stability compared to the same oil processed by different methods such as deodorization, degumming, refining or blenching [23]. Similar results were found for rapeseed oil where the extraction method using hexane as solvent highly affected the oxidative stability of the oil compared to the one obtained by pressing [24]. Recently, Liu et al. [22] investigated the effect of the frying temperature on the unsaturated fatty acids and tocopherols content of ten edible oils. As previously reported by other authors, also in this case, the processing method highly affected the quality of the oils as both tocopherols and fatty acids were degraded [22].

Essential oils are another class of important active components that can benefit from encapsulation technologies. Like vegetable oils, they are obtained from the extraction of herbs and plants. However, the term “essential” highlights the presence of the essence of the plant. Typically, an essential oil contains the most aromatic and characteristic fraction of a plant. Since most of the aroma compounds are sensitive to oxidation reactions, also essential oils need to be protected from external factors, such as oxygen or light [25].

Turek and Stintzing [26] investigated the effect of different storage conditions on lavender, pine, rosemary and thyme essential oils. The degradation of each essential oil was highly dependent on their specific composition. Thyme oil underwent only small modifications. On the other side, rosemary showed a good oxidative stability at room temperature in the dark but oxidized fast in presence of light [26]. Similar results have been published on laurel and fennel oil, which reported a significant decrease of the concentration of their most important compounds such as eugenyl acetate, estragol and transanethole when stored in presence of light suggesting the strong need for both essential oils to be protected using suitable encapsulation technologies [27].

## 4. Supercritical Carbon Dioxide as Encapsulation Solvent

Encapsulation technologies based on supercritical fluids have recently attracted the attention of food industry. As the operations linked to these technologies leave no residues, the use in food processing has great interest to avoid undesirable contaminations [45]. From an industrial point of view, the process is advantageous as it does not involve the use of water or organic solvents. The waste is merely the supercritical fluid, which is naturally present in the atmosphere and can be reused. Additionally, the final product does not have to be purified.

Moreover, thanks to the low temperature needed to turn carbon dioxide into a supercritical fluid, this technology is especially suitable for encapsulating thermo-labile compounds such as vitamins, tocopherols or oils rich in omega-3 polyunsaturated fatty acids. Supercritical fluids are substances at temperature and pressure above their critical point. The most widely used solvent in encapsulation, micronization and particle formation processes is supercritical carbon dioxide (SC-CO_2_) [46,47].

The phase diagram of CO_2_ indicates that the substance reaches the critical temperature at 31.1 °C and the critical pressure at 7.38 MPa. The resulting fluid behaves in between a liquid and a gas. The density is close to that of liquids, while the viscosity is close to that of gases. In addition, SC-CO_2_ has negligible surface tension, which explains its great potential for extraction operations. Moreover, CO_2_ is available in high purity from several sources, it is inexpensive, non-toxic and non-flammable. It has low solubility values in organic solvents and is considered as an environmentally safe solvent with the advantage of being used for developing ecofriendly processes [4,48,49]. A key advantage of SC-CO_2_ over other technologies is its ability to be easily removed upon depressurization, leaving no residue in the sample [1,48,49].

This is a quite important aspect as the produced encapsulated materials are used in foods for human consumption or in pharmaceutical products. Moreover, the high diffusivity, close to the one of a gas, allows the SC-CO_2_ to easily penetrate highly porous nanostructures [4,5,48]. It has a high solubility for non-polar compounds while polar molecules can be dissolved in SC-CO_2_ by adding co-solvents, such as ethanol, methanol and/or other non-polar organic solvents to increase the solubility and enhance the encapsulation process [50].

Overall, all these properties confer to the SC-CO_2_ high attractiveness for applications, which include the processing and production of powder ingredients with desired sizes and functionality to be applied in food matrices [5,49,51,52]. Moreover, the simpler processing steps and the mild operation temperatures achieved during the SC-CO_2_ processes overcome some disadvantages of the traditional encapsulation methods, such as the need of proper cryoprotectants to preserve the bioactive compounds, the numerous steps involved for the preparation and processing of the samples and the high temperatures applied.

## 5. Supercritical Carbon Dioxide Technologies for the Encapsulation of Bioactives

Through the years, different SC-CO_2_ based techniques have been developed based on the nature of the targeted bioactive compound (being soluble or insoluble in SC-CO_2_), the nature of the carrier material and the application of the final microencapsulated compounds [35,41]. In other words, depending on the role played by the SC-CO_2_ in the encapsulation techniques, the CO_2_ can be categorized as a solvent, an anti-solvent, a solute, a co-solvent, an extractor and anti-solvent, an atomization or a drying medium.

Table 2 summarizes the SC-CO_2_ based techniques that are used for encapsulation and for particles formation of active compounds at nano or micro scale for foods applications depending on the role that CO_2_ plays in the process. As an example, if SC-CO_2_ is behaving as a solute, we are dealing with the articles from gas saturated solutions process (PGSS). On the other hand, if SC-CO_2_ acts as an anti-solvent, the gas anti-solvent (GAS) process, a supercritical anti-solvent process (SAS) or a solution enhanced dispersion by supercritical fluid process (SEDS) takes place [38,53,54,55].

In Table 2, information about the encapsulated bioactive compounds, the carrier material, the processing conditions together with the encapsulation efficiency achieved during the process are also reported. In the following paragraphs, each technique is described in depth, providing details about the role played by the SC-CO_2_ in the encapsulation process. Moreover, for each technique, the most significant published studies are reviewed and discussed.

### 5.1. Particles from Gas Saturated Solutions

**Figure 1:**
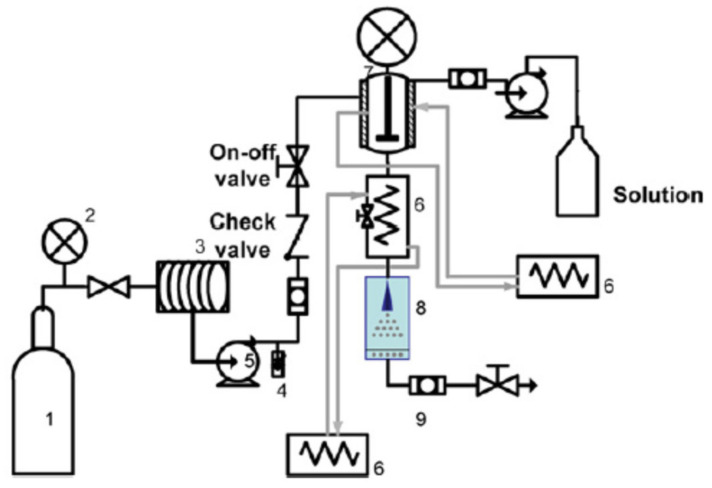
Schematic diagram of gas saturated solutions process (PGSS) technique: 1, CO_2_ tank; 2, pressure gauge; 3, cooling bath; 4, safety valve; 5, pump; 6, heat exchanger; 7, high pressure vessel; 8, separator; 9, filter [61].

The Particles from Gas Saturated Solutions (PGSS) process is the most common example of encapsulation technology based on SC-CO_2_. A schematic diagram simplifying the PGSS process is shown in Figure 1. Briefly, in the PGSS process, the active ingredient and the carrier polymer are both melted in a high-pressure vessel. Then, SC-CO_2_ is let to solubilize into the melt up to saturation. At that point, the solution flows toward a nozzle into a depressurized separator vessel, where particles are formed [119]. Because of its simplicity [5], PGSS is one of the most promising processes for the encapsulation of bioactive compounds for food applications, as indicated by the number of published papers listed in Table 2.

Rodrigues et al. [33] have published one of the first studies dealing with the encapsulation of bioactive compounds by PGSS technique. The authors produced new carriers of theophylline prepared with hydrogenated palm oil with controlled-release properties. The PGSS system used for the experiments was equipped with a mixing vessel where the theophylline and the hydrogenated palm oil as carrier material were loaded. A temperature of 60 °C was chosen to melt the palm oil. SC-CO_2_ was pumped from the storage cylinder into the mixing vessel through a nozzle until reaching the desired operative pressure. After a defined processing time, the liquid mixture was precipitated by opening an expansion valve and rapidly expanding the solution to atmospheric pressure through a small orifice inside a stainless-steel tube. The particles produced were characterized by two principal morphologies: large spherical particles and small needle- (or fibrous-) shaped particles. Overall, the particles presented a morphology resembling spheres with spikes. The mean particle size resulted in the range 2.5–3.0 μm and containing from 0.5% to 3.5% of theophylline. The in vitro release study revealed that about 22–45% of the total theophylline content initially present on the particles was mainly located at the surface of the particles as it was quickly released. The remaining content of the drug was encapsulated in the inner core of the particles indicating that it could be protected from degradation, premature elimination and consequently released in a controlled way [33].

After this first study, the literature is abundant on PGSS findings showing the potential of the technique for the micronization of food ingredients and bioactive compounds such as mackerel reaction oil [66], menthol [70], β-carotene [30] and several essential oils [39,59,71,75]. In most of the studies, polyethylene glycol has been used as carrier material for the encapsulation and protection of bioactives.

As an example, Ndayishimiye and Chun [71] investigated the encapsulation of citrus oil by PGSS process using polyethylene glycol. Particles with sizes in the range from 190.56 to 373.32 μm and different morphologies were obtained with an encapsulation efficiency ranging between 44% and 84%. In addition, the oxidative stability of the citrus oil was significantly improved by the PGSS encapsulation [71].

Getachew and Chun [67] optimized the coffee oil flavor encapsulation by PGSS process obtaining a maximum encapsulation efficiency of 80%. The encapsulated oil reported a peroxide value equal to 4.56 meq peroxide/kg oil after 12 weeks of storage. This result indicated that less than 2% loss of fatty acid composition after encapsulation was lost. Moreover, the powdered ingredient showed a very good preservation of flavors. Therefore, it was concluded that PGSS microencapsulation could be used to produce free flowing powdered ingredients suitable for food industries [67].

Several studies also claimed the possibility to obtain particles of different sizes and morphologies changing the nozzle type or the processing conditions. In this vein, it is possible to obtain the formation of micro or nanoencapsulated bioactive compounds. Microencapsulation is used to obtain solid particles with specific properties, environmental protection and controlled release characteristics of bioactive compounds having diameters between 1 to 1000 µm [120]. On the other side, nanoencapsulation is defined as the technology to encapsulate substances at the nanoscale range with the potential to enhance bioavailability and improve the controlled release in a greater extent than microencapsulation as the produced nanoparticles have diameters ranging from 10 to 1000 nm [121]. Indeed, thanks to the tunable properties of the SC-CO_2_, it is possible to move from micro to nanoscale.

As an example, in the study of Haq and Chun [31], micrographs of astaxanthin rich salmon oil microparticles encapsulated in polyethylene glycol were obtained by PGSS. The microparticles reported an irregular shape with different morphologies from spherical to amorphous with different sizes. More agglomerated and bigger microparticles were obtained with nozzles of high diameters (400 and 500 μm) compared to those obtained using a smaller nozzle of 300 μm. This was associated to the higher amount of free oil on the surface of the microparticles, which bound them together by capillary forces [31].

**Figure 2:**
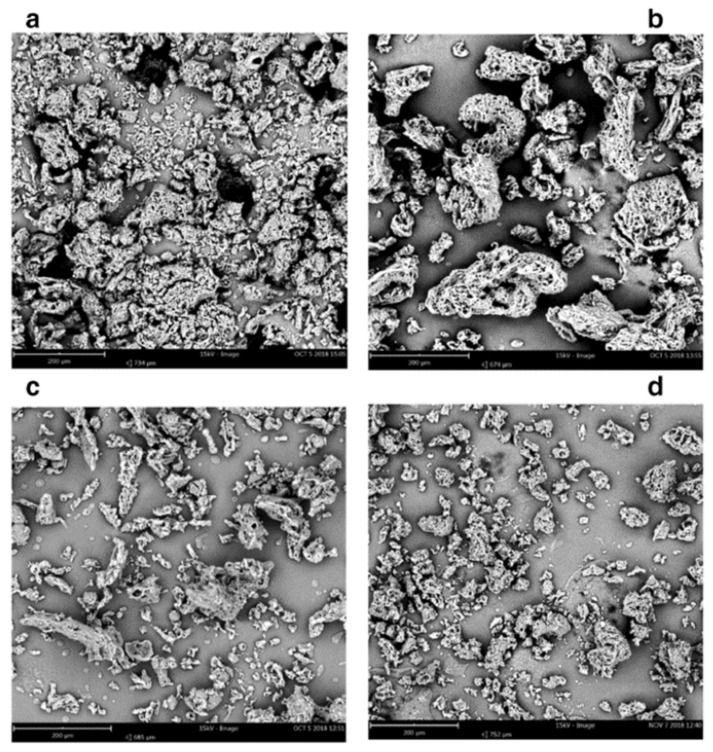
Scanning electron micrographs of microparticles of oils recovered from brewer’s spent grain were encapsulated in polyethylene glycol by PGSS. From (**a**–**d**) increased processing pressure: (**a**) 10 MPa, (**b**) 20 MPa, (**c**) 30 MPa, (**d**) 35 MPa [75].

The effect of the processing pressure on the morphology of the microparticles was investigated in the study of Ndayishimiye et al. [71] where oils recovered from brewer’s spent grain were encapsulated in polyethylene glycol by PGSS. They observed a clear different morphology and particle size changing the pressure from 10 to 35 MPa as shown by the scanning electron micrographs of Figure 2. Among the functional properties of the microencapsulated oil, they studied the oxidative stability. The PGSS produced samples were oxidative stable up to 360 h at 50 °C achieving a shelf life four times longer compared to the not encapsulated oil [71].

An interesting study has been published for the encapsulation of Vitamin B2 in solid lipid nanoparticles by using a modified PGSS process [36]. The process was modified by performing the decompression in a water stream, instead of air or nitrogen, to produce nanoparticles of more uniform shape and smaller size. The authors were able to produce nano-scale solid lipid particles with a content of hydrophilic bioactive of 0.54 ± 0.05 mg/g in polyethylene glycol with a bimodal particle size distribution. They concluded that the modified PGSS process was able to produce hydrophilic bioactives encapsulated in solid lipid nanoparticles in line with those found in the literature employing other techniques. However, further works were needed involving a full characterization of the obtained particles, including crystallinity, morphology and stability, in order to assess the full potential of the process.

### 5.2. Particles from Gas Saturated Solutions Drying

The Particles from Gas Saturated Solutions Drying (PGSS-Drying) technique is a modification of the PGSS process. Like in the PGSS process, PGSS-Drying includes a static mixer, which is used to intensively mix the carrier material, the active ingredient and the SC-CO_2_. However, unlike PGSS, the carrier material and the active ingredients are firstly dissolved in a considerable amount of solvent (i.e., water). Then, this solution is saturated with SC-CO_2_ and sprayed into an expansion chamber, where CO_2_ turns into gas. However, here, because of the excess of solvent, fine droplets are formed, which turned into powder evaporating the solvent by increasing the temperature in the expansion chamber. Ideally, the precipitation of the powder is carried out in an oxygen-free atmosphere, avoiding any side reaction which may occur to sensitive substances [52,122].

In 2000, Weidner [123] patented for the first time the PGSS-drying process. However, he wrote the first scientific publication on the process some years later in 2008 reporting results on the drying of aqueous green tea extracts [76]. Since then, PGSS-drying has been successfully applied for bioactives encapsulation for food applications. Thanks to the presence of the drying step, also water-soluble carrier materials such as starch, maltodextrin and lecithin can be used for the microencapsulation [78,81,83].

Varona et al. [38] encapsulated lavandin essential oil in n-octenyl succinic modified starches by PGSS-drying technique to produce a biocide to use for the agriculture. In a subsequent study, soybean lecithin was used as carrier material to encapsulate β-carotene giving spherical particles of sizes ranging from 10 to 500 µm with an encapsulation efficiency equal to 60% [78].

It is worth to mention the recent study published by Melgosa et al. [83] where for the first time omega-3 polyunsaturated fatty acids were encapsulated by PGSS-drying. A comparison was then carried out with the microparticles obtained by conventional drying methods such as spray-drying and freeze-drying. A spherical morphology was observed for the PGSS-dried powders like the one obtained by spray-drying, while freeze-drying produced powders with irregular morphologies. In addition, the encapsulation efficiency of the PGSS and spray-dried powders was comparable and equal to about 98%. Compared to the conventionally dried powders, PGSS-dried microparticles reported 28 days of storage at 4 °C with low concentration of primary and secondary oxidation products. These results highlighted the superior ability of PGSS-drying to produce ingredients with enhanced functional properties [83].

### 5.3. Rapid Expansion of Supercritical Solutions

Rapid Expansion of Supercritical Solutions (RESS) is a technology quite similar to PGSS. However, here the solute is dissolved in SC-CO_2_ [45]. Briefly, in the RESS process, SC-CO_2_ is continuously flowing to the extraction chamber, where solids substances are placed. During the flow through the solids, some of the solutes are solubilized into the SC-CO_2_ stream. This solution is conveyed into a low-pressure chamber and forced to pass through a heated nozzle. The sudden expansion of SC-CO_2_ causes a rapid cooling and drop of the pressure. This leads to the collapse of solutes into particles.

Particles produced by RESS are usually much smaller than those obtained by PGSS with a uniform morphology due to the high supersaturation ratios achieved during the process. However, by changing the processing parameters such as the temperature, pressure and nozzle geometry, larger particles can be produced. The number of studies on the application of RESS in the area of food products is quite scarce due to the limited or moderately solubility of some food grade compounds in SC-CO_2_. As an example, to get the solubilization in CO_2_ of 1% of a triglyceride, a pressure higher than 10 MPa is required. For other substances, like carotenoids, even lower concentrations of about 1 order of magnitudes lower can be achieved [5]. Studies applying RESS techniques for the encapsulation of bioactive compounds as ingredients for the food industry are reported in Table 2.

Santos et al. [88] studied the encapsulation of anthocyanin extracted from jabuticaba skins using polyethylene glycol as a carrier material. The effect of processing variables (pressure, temperature and core material to polyethylene glycol ratio) on the encapsulation efficiency was also investigated. The results showed that the encapsulation efficiency was a strong function of the pressure. This was attributed to the increase of CO_2_ density with the pressure. Moreover, the antioxidant activities of the encapsulated and non-encapsulated extracts were compared to evaluate the efficiency of RESS process. The encapsulated anthocyanin extracts retained a dark red color, indicating that probably there was no significant degradation during the encapsulation procedure, and the encapsulated extract showed higher oxidative stability than the non-encapsulated ones highlighting the role of RESS process [88].

However, the main limitation of RESS is linked to its difficulty to encapsulate high polar compounds or compounds with low solubility in SC-CO_2_. This aspect dramatically affects its application. To overcome this limitation, research studies proposing some modifications to the technique have been developed. They require the possibility to employ alternative organic supercritical solvents such as trifluoromethane or clorodifluoromethane [45].

As an example, a valid modification of the process consists in using a liquid anti-solvent as co-solvent for improving the solubility in the supercritical fluid. This modified process has been defined as RESS-non-solvent process (RESS-N). It has been rarely applied for the encapsulation of food bioactive compounds due to the presence of organic co-solvents, usually not food grade.

One of the few papers published so far using the RESS-N has been carried out to form polymer microparticles containing proteins such as lysozyme and lipase. The study was performed by preparing a mixture of proteins in CO_2_ containing a co-solvent and a dissolved polymer. Different polymers were tested such as polyethylene glycol, poly(methylmethacrylate), poly(L-lactic acid), poly(DL-lactide-co-glycolide), and poly(propylene glycol). The mixture is sprayed through a nozzle in a vessel reaching the atmospheric pressure. The authors demonstrated that by knowing the phase equilibria of the mixture, it was possible to produce polymeric microcapsules without any agglomeration with monodisperse size and a particle size distribution controlled by changing the polymer feed composition. The morphology and particle size distribution of the obtained powders were not affected by the pressure, temperature, molecular weight of polymer, and injection distance of the mixture inside the vessel [93].

### 5.4. Gas Anti-Solvent Process

The gas anti-solvent (GAS) process is a precipitation technique, which produces powders at high yields with narrow size distribution. The GAS process is straightforward. It is based on the capacity of SC-CO_2_ to remove the organic solvent in which the bioactive is dissolved. The removal of the organic solvent induces the precipitation of the bioactive. Accordingly, the main critical aspect of the process is that SC-CO_2_ needs to have a high solubility in the organic solvent but a very low solubility with the bioactive [124]. Consequently, this technique is especially suitable for polar compounds like proteins and peptides since they are usually not soluble in SC-CO_2_ [4].

The GAS process is carried out in batch or discontinuous mode. The typical operations of the GAS process start with the bioactive compound and a wall material dissolved in an organic solvent. The active solution is then filled with CO_2_. Then, the temperature and pressure of the system are increased until the supercritical conditions are reached [124]. In a supercritical state, also the volume of the organic solvent is expanded. Because of the organic solvent evaporation, both the bioactive compound and the carrier material precipitate. SC-CO_2_ and the expanded organic solvent are then discharged, while the particles are trapped in a crystallizer vessel. CO_2_ gas is then recovered in a separator vessel and the organic solvent drained. The recovered CO_2_ can be then flushed over the microparticles for a final drying step.

**Figure 3:**
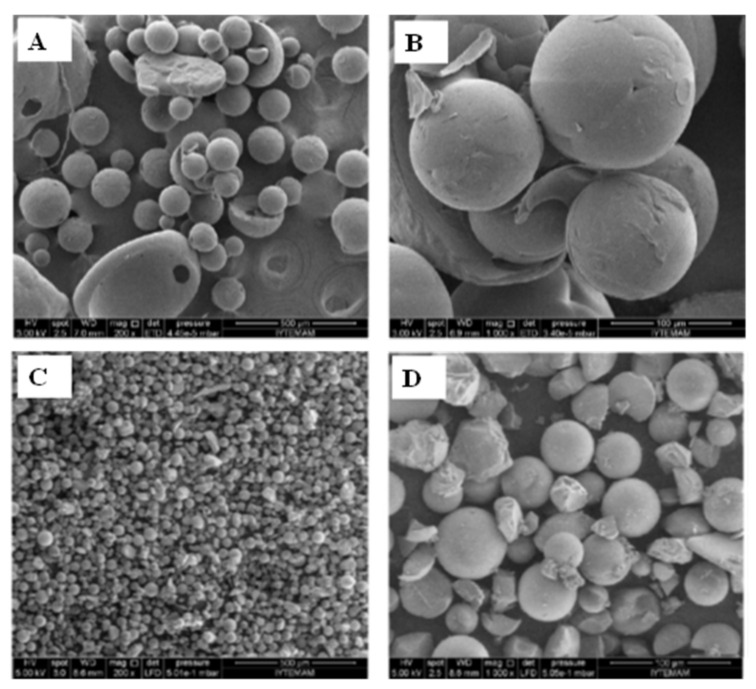
Scanning electron micrographs of polycaprolactone alone (**A**, **B**) and encapsulated with rosemary extract by GAS (**C**, **D**). Adapted from Yesil-Celiktas and Cetin-Uyanikgilb [54].

Yesil-Celiktas and Cetin-Uyanikgilb [54] published a study based on GAS to produce microparticles of rosemary extract encapsulated in polycaprolactone. In this example, the rosemary extract and the polycaprolactone were both dissolved in dichloromethane. Then, CO_2_ was pumped into the vessel. Its anti-solvent effect caused the reduction of the solvent power of dichloromethane. Consequently, the remaining solution became supersaturated, leading to the formation of encapsulated particles. Moreover, the particles were washed with SC-CO_2_ to remove the remaining dichloromethane. Overall, the resulting efficiency of the process was very high (83%). Moreover, a mean particle size of 255 nm was achieved with a narrow size distribution. The morphologies of the produced ingredients are reported in Figure 3 C, D where their spherical structure, smooth surface and absence of agglomeration are visible [54].

### 5.5. Supercritical Anti-Solvent Process

Supercritical anti-solvent process (SAS) also uses the principle of anti-solvent technique. Similar to the GAS process, in SAS the SC-CO_2_ still acts as anti-solvent. However, the contacting mechanism is different as SAS process is carried out in semi-continuous way with the continuous delivery of solvent and anti-solvent in the precipitator. In SAS process, the liquid CO_2_ is first fed into the precipitation vessel and pressurized. Then, it is heated to the desired temperature. When the system reaches equilibrium, the mixture comprised of the bioactive compound, the wall material and the organic solvent is injected into the precipitation vessel [125]. This technique has been extensively used for the encapsulation and production of micronized particles for food, polymer and pharmaceutical applications [53,92,126,127,128,129,130].

Specifically in food applications, Chinnarasu et al. [131] encapsulated antioxidants from *Olea europaea* leaves using SAS process and investigated the role of SAS process to stabilize those antioxidants. From their results, it was remarkable that not only the SAS process stabilized the antioxidants of *Olea europaea* leaves but also most of the compounds from the extract were preserved after the SAS process [131].

Some years before, antioxidants from rosemary leaves [53] and polyphenols from green tea [99] were successfully encapsulated by SAS. Both studies indicated that a high encapsulation efficiency was reached with products showing small particle sizes with narrow distribution and a high degree of agglomeration.

**Figure 4:**
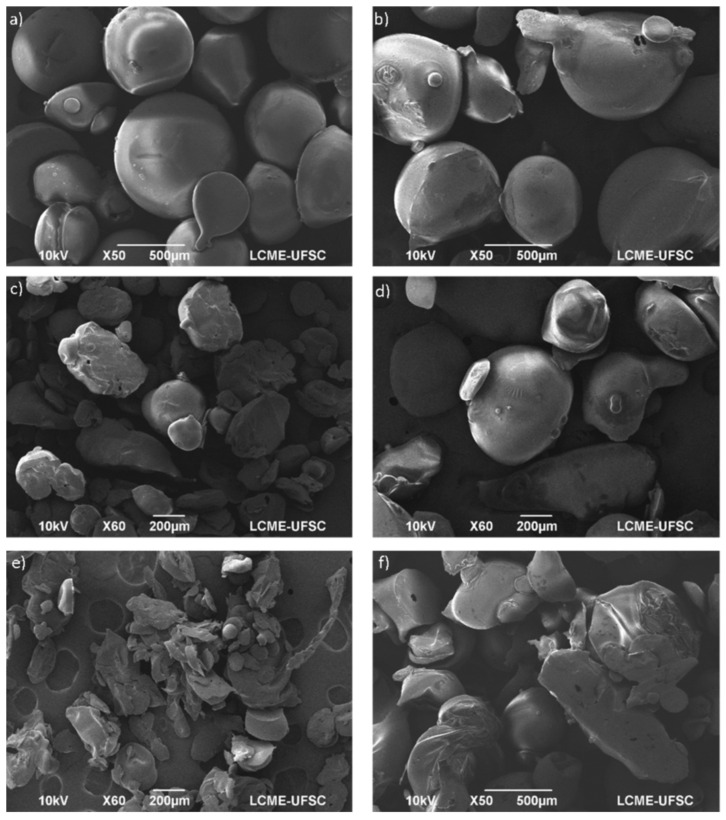
Scanning electronic microscopy images for passion fruit seed oil encapsulated in (poly(lactic-co-glycolic) acid) by SAS. Images (**a**–**f**) were for powders obtained at different processing conditions [102].

Recently, Oliveira et al. [102] recovered oils from passion fruit seeds. The recovered oils were then encapsulated using a biopolymer (poly(lactic-co-glycolic) acid) as carrier material by SAS process. As shown in Figure 4, the particles morphology and size varied from spherical shape at low pressure to irregular shape at high pressure. The particle size ranged from 721 to 1498 nm with an oil encapsulation efficiency changing from 68% to 91% [102].

Few studies have been published so far testing the functional properties of the encapsulated bioactive compounds by SAS process. The main feature that was taken into account for such microparticles was their dissolution behavior in simulated gastric and intestinal fluids. This aspect was tested on quercetin encapsulated in Pluronic F127 poloxamers [101], polyphenols extracted from green tea and encapsulated in poly-ε-caprolactone [99] and antioxidants from rosemary encapsulated in Pluronic F88 and Pluronic F127 poloxamers [53].

In detail, Fraile et al. [101] reported the study of the released trials of quercetin in simulated gastric and intestinal fluids. A faster dissolution and a higher solubility (4 times higher) of the encapsulated quercetin were observed, compared to the product obtained by a simple physical mixture of the compounds. The increased dissolution was associated to the very small particle size obtained after the SAS process [101].

On the other side, Sosa et al. [99] studied the release behavior of the encapsulated polyphenols from green tea in phosphate buffer at pH of 6.8. They observed that 30% of the encapsulated compounds was released after about 90 h while in the first 4 h an amount equal to 15% was dissolved in the buffer. The remaining amount of polyphenols was tightly crystallized inside the polymeric matrix and further released only when the polymer was degraded. This process may occur in months as supported also by the results of the differential scanning calorimetry measurements carried out on the same samples [99].

Similarly, Visentin et al. [53] investigated the release behavior of antioxidants extracted from rosemary and encapsulated in a mixture of biopolymers in an aqueous medium. Their results reported that after 1 h about 100% of the total polyphenolic content was dissolved from the encapsulated matrix while about 65% of the polyphenols were dissolved from the pure extract mixed with the biopolymers as surfactants and only 3% of the polyphenols from the pure extract. They also reported a lower degradation of the encapsulated compounds (50% less) compared to that one achieved for the polyphenols just physically mixed with the biopolymers. In conclusion, their findings indicated on one side a faster dissolution kinetic while on the other side a better protection against degradation factors for the compounds encapsulated by SAS [53].

As concerns the application of SAS for the encapsulation of vitamins, just one study has been published. In detail, Fei et al. [98] showed the possibility to produce proliposomes made of hydrogenated phosphatidycholine and vitamin D_3_ testing the effects of processing conditions such as temperature, pressure and components on vitamin D_3_ entrapment in the final product. They reported the formation of nanospheres of proliposomes of vitamin D_3_ obtained at the optimum conditions of 8 MPa, 45 °C and 15% mass ratio between the vitamin and the hydrogenated phosphatidycholine. The authors also compared the particles obtained by SAS with those produced by a thin-film and ultra-sonic dispersion method. The results indicated a higher entrapment efficiency of the vitamin for the particles obtained by SAS. They reached a value of 100% of entrapment efficiency thanks to the procedure applied to have the proliposomes by SAS [98].

### 5.6. Solution Enhanced Dispersion by Supercritical Fluid

**Figure 5:**
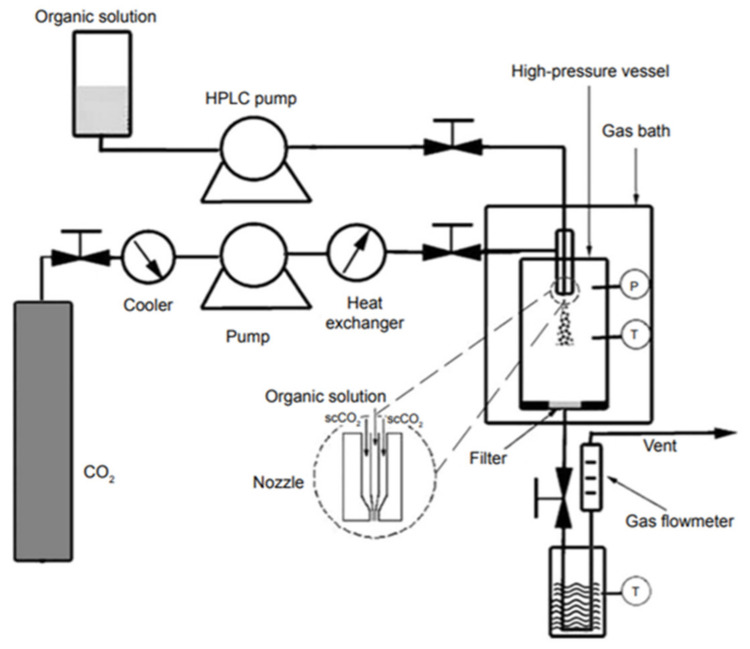
Schematic diagram of SEDS process. Abbreviations: SEDS, solution-enhanced dispersion by supercritical carbon dioxide (CO_2_); P, pressure; T, temperature; HPLC, high performance liquid chromatography [134].

The solution enhanced dispersion by supercritical fluid (SEDS) is a modified version of the SAS process [55,110] where a specially designed coaxial nozzle is used to spray the mixture of the bioactive compound, wall material, solvent and SC-CO_2_ (Figure 5). In this process, the SC-CO_2_ can have several purposes. It can be used not only as an anti-solvent but also as a dispersing agent. The contact of a solution containing the bioactive compound and the carrier material with the SC-CO_2_ can produce a finely dispersed mixture, which will then precipitate. SEDS can be also applied on aqueous solutions forming microencapsulated particles from water-soluble compounds such as proteins and sugars [132]. Moreover, SEDS has another great advantage for encapsulation as it is designed also for carrier materials and bioactive compounds that are not soluble in the same solvent. To perform this process, two different solutions with each of the substances can be prepared and then subject them simultaneously to SEDS precipitation. The nozzle needs to be properly designed to allow the simultaneous injection of the two liquid solutions into the SC-CO_2_ [133].

**Figure 6:**
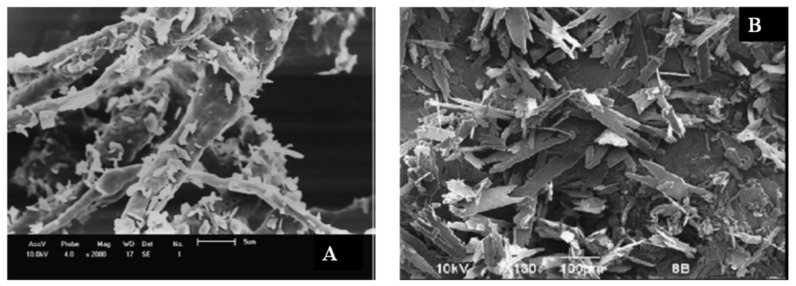
Scanning electron micrographs of β-carotene encapsulated in poly (3-hydroxybutirate cohydroxyvalerate) by SEDS at different magnification ((**A**), 5 μm; (**B**), 100 μm) [105,106].

Nanoparticles formation of lycopene/β-cyclodextrin was carried out via SEDS. N, N-dimethylformamide and SC-CO_2_ were used as solvent and anti-solvent, respectively. The process produced small spherical particles. In detail, it was possible to obtain particles with an average particle size of about 40 nm applying high pressures (14 MPa), high temperatures (50 °C), high CO_2_ flow rate (0.75 mL/min) and low solution flow rate (15 mL/min) [135]. In further studies, the encapsulation of β-carotene and poly (3-hydroxybutirate cohydroxyvalerate) via SEDS process was also investigated [105,106]. In both studies, it was highlighted the strong effect of pressure on the particle size and morphologies of the obtained powders. Indeed, by increasing the pressure from 8 to 12 MPa leaf like particles were obtained (Figure 6).

Recently, natural grape seed extracts [107] and pink pepper extracts [108] have been encapsulated by SEDS using poly (3-hydroxybutyrate-co-3-hydroxyvalerate) as carrier material and dichloromethane as solvent. Grape seed extracts, encapsulated at 8 MPa, 35 °C and extracts to carrier material mass ratio of 1:1, led to spherical particles of about 0.70 μm and encapsulation efficiency of 66% [107]. Similarly, depending on the processing conditions, microparticles of encapsulated red pink pepper extracts reported spherical shapes, average diameters from 0.39 to 25.4 μm and encapsulation efficiency from 20% to 95% [108].

As concern the functional properties of SEDS microencapsulated compounds, no studies have been performed so far. Most of the published works aimed to optimize the processing parameters to reach a specific encapsulation efficiency and investigate the possibility to obtain encapsulated compounds with a defined morphology and particle size distribution. Indeed, further experimental studies are needed to prove that the technology can be applied to produce encapsulated compounds for food applications.

### 5.7. Supercritical Fluid Extraction of Emulsions

Supercritical fluid extraction of emulsions (SFEE) process uses CO_2_ as an extractor and anti-solvent for encapsulation and production of microcomposites. The process has been designed to carry out using SC-CO_2_, the conventional process that produces particles starting from an emulsion by evaporation of the solvent or by extraction using a second solvent [136]. The process consists of two parts. A first part deals with the formation of an emulsion. The second part, instead, deals with the extraction of the organic solvent from the emulsion.

Usually, the process starts with the preparation of an emulsion by dissolving the bioactive compound and the carrier material in a suitable organic solvent. The solution is then mixed with water forming an oil in water emulsion. A surfactant is often added as stabilizer. The emulsion is then sprayed in a vessel purged continuously with SC-CO_2_. The SC-CO_2_ results highly miscible with the organic solvent having the functions of both an anti-solvent and an extraction fluid at the same time [45]. It extracts the oily (organic) solvent from the suspension through the water or directly when is in contact with the organic phase. Moreover, the CO_2_ diffuses into the small solvent droplets acting as an anti-solvent causing the supersaturation of the mixture. The precipitation of the particles suspended in the water phase and stabilized by the surfactant is caused by the fast extraction of the organic solvent and the anti-solvent effect of the SC-CO_2_. At the same time, the mixture of CO_2_ and organic solvent is continuously removed from the system.

Many works have been carried out to study the application of SFEE for the production of encapsulated compounds in foods [82,114,137,138,139]. Prieto et al. [137] used SFEE to encapsulate fish oil rich in omega-3 polyunsaturated fatty acids in polycaprolactone as carried material. Three emulsion formulations containing different stabilizing agents were tested. They comprised Tween 80 as a surfactant, polycaprolactone as a coating polymer and acetone as an organic solvent. Based on the formulation, it was possible to obtain spherical and non-aggregated nanoparticles with sizes ranging from 6 to 73 nm. The nanoparticle encapsulation efficiency produced by SFEE was around 40%. The same result was achieved by conventional solvent evaporation. By performing the SFEE at 8 MPa and 40 °C, about 25 kg CO_2_ per kg of acetone were needed to reduce to 5000 ppm the acetone concentration in the encapsulated particles. This was the requirement needed to use the product for pharmaceutical application. However, for food applications, the allowed maximum acetone was decreased to 50 ppm. To achieve this requirement, the CO_2_ consumption was increased to about 127 kg CO_2_ per kg of acetone.

Reátegui et al. [140] produced copaiba (*Copaifera officinalis*) oleoresin particles using SFEE. A modified starch was used as carrier material. Ultrasounds were used to produce the oil in water emulsions by mixing the oleoresin with ethyl acetate, modified starch and water. The efficiency of the process was evaluated in terms of residual ethyl acetate content and β-caryophyllene recovery, which was the target compound quantified in copaiba oleoresin. SFEE was able to achieve about 94% of ethyl acetate removal. The residual ethyl acetate content was within the exposure limit (5000 ppm/day). The β-caryophyllene recovery was equal to 7.3% while the size of the suspended encapsulated particles was about 260 nm [140].

Overall, all the studies published so far claimed the advantages of SFEE technique to provide microencapsulated compounds with high encapsulation efficiencies, narrow particle size distribution with spherical and non-aggregated morphologies [82,137].

An interesting study has been published comparing the microparticles obtained by SFEE with those obtained by SAS. The authors recovered carotenoids from pink shrimp residues, which were then encapsulated by SFEE and SAS. The emulsion was prepared mixing the extract with acetone and using Pluronic F127 for SAS and a modified starch for SFEE as carrier materials. The highest encapsulation efficiency was achieved by SFEE reaching about 93% while SAS produced microparticles with an encapsulation efficiency equal to 74%. Moreover, SFEE yielded particles with nanoscale dimensions and size ranging from 0.8 to 7 mm [100].

Recently, Levai et al. [82] produced microparticles of quercetin encapsulated by SFEE then subsequently dried by PGSS. A comparison was also carried out by drying the product by freeze-drying. The emulsion was prepared by dissolving quercetin in soybean lecithin as surfactant and Pluronic L64 as carrier material. PGSS-drying provided microparticles with the same antioxidant activity, no quercetin degradation and similar encapsulation efficiency as those produced by freeze-drying confirming the suitability of the process. Moreover, the PGSS dried quercetin microparticles reported an enhanced permeability through the transdermal membrane into a simulated intestinal fluid compared to the freeze-dried microparticles. This conferred a higher potential to the microparticles produced with the combination of SFEE and PGSS-drying in terms of functional properties [82].

### 5.8. Liposomes Formation by Supercritical Fluids

Recently, supercritical fluid-based techniques have been also proposed for the production of liposomes as alternative to the conventional encapsulation methods (i.e., thin film hydration, ethanol injection and reverse phase evaporation or detergent dialysis methods). In most of the conventional methods, the starting point for liposomes production is the dissolution of phospholipids in an organic solvent. Then, the lipid membrane is dispersed in an aqueous medium and dried [141,142,143].

Through time, the use of SC-CO_2_ for the production of liposomes has been slightly modified moving from two steps processes, in which dried lipid particles are obtained and then rehydrated to obtain the liposomes, to one step processes, in which a liposome-water suspension is directly obtained at the end of the process. Works published in recent years evidenced some evolution of the two approaches by producing first water based micro- and nanodroplets and then by forming the liposomes around them. The idea is that lipids contained in the expanded liquid can spontaneously organize in a layer around the water droplets in the high-pressure vessel forming a water in CO_2_ emulsion. At the end of the process, the droplets fall in a water pool placed at the bottom of a vessel. In this way, water in water emulsion can be formed [117,144].

**Figure 7:**
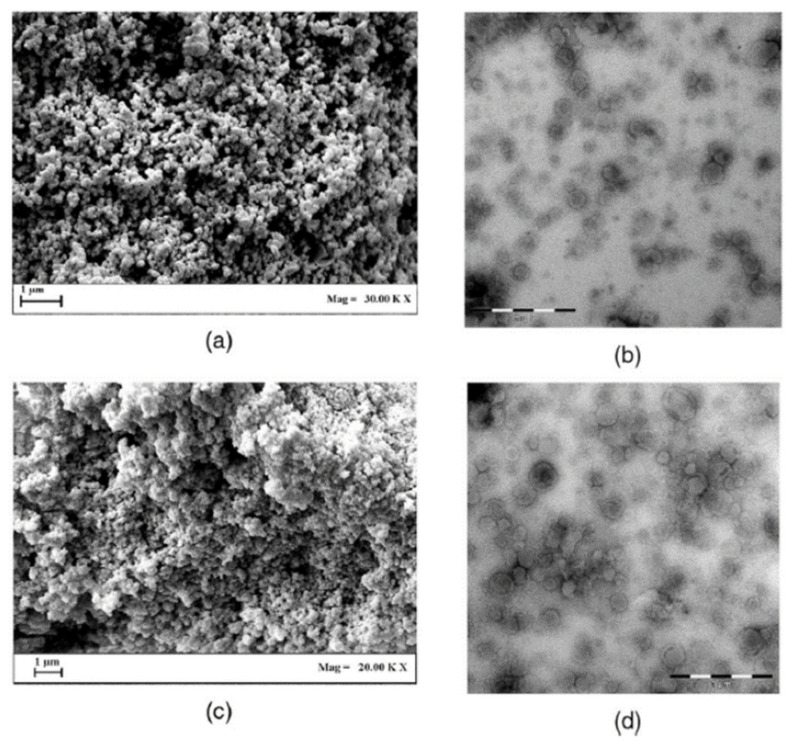
Scanning electron microscope ((**a**,**c**) at different magnification) and transmission electron microscope ((**b**,**d**) at different magnification) images of liposomes loaded with eugenol processed at different conditions [118].

Studies showed the efficiency of supercritical fluid techniques in producing liposomes suitable for food application by encapsulating lutein [46], anthocyanin [116], proteins [117] and eugenol [118]. The scanning and transmission electron images, reported in most of the abovementioned works, report liposomes with unilamellar, spherical or near spherical shapes. An example is shown in Figure 7.

Moreover, the produced liposomes presented size and distribution of dimensions in nanometer scale. As an example, liposomes with eugenol reported a mean diameter of about 230 nm [118], while liposomes of soybean phosphatidylcholine with proteins reported distributions ranging between 250 and 330 nm [117].

Similar results were also achieved by Zhao et al. [116] who also addressed for the first time the rate of release of the anthocyanin from liposomes in simulated gastric and intestinal fluids. Their results indicated a slow release of anthocyanin from liposomes in the simulated gastric fluid, which became faster in the intestinal fluid due to the degradation of the vesicles by pancreatin. The authors claimed the need to perform further studies, possibly acting on surface modifications of the liposomes by adding an additional chitosan coating layer, to enhance the stability in the small intestinal tract and improve the functionality of the liposomes for functional food applications [116].

## 6. Industrial Scale Applications

So far, the studies published and the results achieved for some of the supercritical fluid based techniques clearly highlight their potential transferability at industrial scale. Indeed, some of the processes dealing with the anti-solvent or the solute role of SC-CO_2_ are in use by some companies working in the field of food science and technology [5].

As an example, a modified anti-solvent process has been applied for the production of lecithin in powder. The process consists of spraying a liquid mixture containing raw lecithin with about 40% of oil through a nozzle in a pressurized vessel with SC-CO_2_. The oil has the function of an anti-solvent being able to be solubilized in the CO_2_ while the lecithin precipitates in powder form. The technology has been patented by Uhde GmbH (Germany) [145] and is used at industrial scale by Jiusan Group in China producing about 600 tons of lecithin per year [1,5,146].

As concern the PGSS-type process, its transferability at industrial scale has been much easier due to the relatively lower investment and operating costs. Moreover, the process is based on the solubilization of supercritical CO_2_ in oils and fats, which can dissolve around 30% of CO_2_ at pressures of 10 MPa. As shown by the results of the published studies, this high amount of solubilized CO_2_ is able to reduce the melting point of the mixture and make it able to be spray dried in an expansion vessel through a nozzle.

Thanks to these advantages, in 2008, an industrial PGSS plant started to be operative in Oberhausen (Germany) at the Fraunhofer Institute UMSICHT with the capacity to produce up to 300 kg of powdered ingredients per hour working with a maximum pressure of 35 MPa and a temperature of 200 °C [5]. In Germany also, the company NATECO_2_ flanked an industrial PGSS system to produce food ingredients to an extraction plant operating with SC-CO_2_ [147].

Recently, Unilever in collaboration with FeyeCon (Weesp, The Netherlands) has made the first steps in the improvement of the environmental footprint of their spreads portfolio made of butter, cream and margarine using a new technology called “Cool blending” based on solid lipid particles micronized by SC-CO_2_ [1,148].

Another technique based always on supercritical fluids and called Pressurized Gas Expanded technology (PGX) has been implemented at industrial scale by Ceapro Inc. [149] in Canada. The method involves the use of CO_2_ and ethanol for water removal and the precipitation and impregnation of bioactive compounds with high molecular biopolymers.

**Figure 8:**
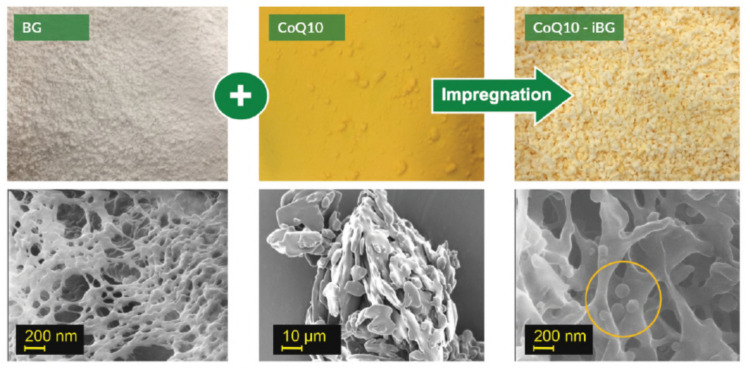
Processed oat beta glucan (BG) by PGX with the addition of the co-enzyme Q10 to produce an encapsulated ingredient (CoQ10-iBG) [85].

In 2017, the company successfully developed a new water-soluble chemical complex composed of co-enzyme Q10 and oat beta glucan (Figure 8). Moreover, new tablets of oat beta glucan were produced with functional properties able to reduce the cholesterol as assessed by some trials performed on human clinical tests [85,86,150,151].

## 7. Future Perspectives and Final Remarks

The studies published on the use of supercritical fluid-based techniques indicate that different types of encapsulated bioactive compounds can be produced. However, several aspects need to be considered before moving towards an industrial implementation and a subsequent production of the ingredients.

The first aspect to consider is that the encapsulation and particles formation using SC-CO_2_ is primarily driven by the solubility or insolubility between the active compound and the carrier material in CO_2_ at the supercritical state. This represents the main drawback and one of the limiting factors of supercritical encapsulation techniques. The solubility of the active compound in the carrier material indicates the limit of concentration of the active compound reached during the encapsulation. Moreover, solubility data also indicate the amount of bioactive that can be effectively encapsulated forming a solid product, rather than a dispersion of segregated particles of carrier and bioactive. Thanks to the knowledge of these data, the shelf-life of formulations can be also assessed since products with concentration of bioactive in the carrier material higher than the equilibrium are likely to undergo degradation processes by segregation of the active compound out of the carrier. As an example, de Paz et al. [152] published solubility data of β-carotene in the range of temperature between 10 and 50 °C in poly-(ɛ-caprolactones) of different molecular weights produced by SFEE technique. They observed that the solubility data of the active compound were highly dependent on the molecular weight of the carrier material and that the temperature of the process significantly affected the solubility. They were able to identify the optimum temperature and the best molecular weight of the polymer to carry out the encapsulation by SFEE [152]. A similar approach was previously proposed also by Kluge et al. [138,153] who provided solubility data of Ketoprofen on poly(lactic-co-glycolic acid) by SFEE encapsulation. However, the studies published so far are really few. More information is needed to gain knowledge about miscibility and solubility data of bioactive compounds and carrier materials to design the optimum formulations [138,153].

Another drawback of these techniques is linked to the solvents used, which are not food grade. As an example, in the anti-solvent processes, the solute of interest is first dissolved in a conventional organic solvent (ethanol, methanol, acetone, dichloromethane) and then put in contact with the SC-CO_2_ with the role of an anti-solvent. The SC-CO_2_ should be miscible with the organic solvent but immiscible with the solute. This aspect represents a serious limitation for the applicability of processes such as SAS, SEDS or SFEE for food products.

On the other hand, for processes such as PGSS, PGSS-drying or PGX, the main drawback is the choice of the carrier material. In most of the published studies, synthetic polymers have been used. However, in most of the applications in use by the food industries, bioactive compounds are encapsulated in carrier materials such as polysaccharides, starches, modified starches, β-cyclodextrin or whey protein, which are considered as food grade ingredients.

As a final remark, the physicochemical and functional features of the final powders represent an important aspect to consider. From a detailed analysis, the lack of knowledge and predictability linking the functional properties of the ingredients produced by the supercritical fluid based techniques with the processing parameters ruling the particles formation and the subsequent application in a real food product are quite evident.

As an example, the cited studies show the several possible ranges of particle sizes and morphologies that can be reached by using such techniques. Nevertheless, the information obtained so far is mainly used to understand and control the effect of the processing parameters on the morphology of the produced food ingredients. However, no studies have been published so far applying the ingredients in real food products with the aim to understand if the claimed obtained physicochemical and functional properties are transferred to them. These considerations indicate clear directions and perspectives that the research dealing with supercritical fluid-based encapsulation technologies needs to address in future studies.

In conclusion, encapsulation techniques based on the use of SC-CO_2_ have received increasing attention thanks to the ability of the solvent to produce ingredients with desired functional properties. Although some of these techniques already found their application at the industrial scale, more studies are needed to improve the processes through the optimization of the processing variables to obtain standardized encapsulated bioactives. The published data demonstrate that these techniques have some advantages over the existing conventional ones. However, the fundamental aspects of the formation of micro and nanoparticles seem still obscure for some of the processes. This aspect clearly indicates that an extensive research is required to reduce the limitations linked to the understanding of the functionalities of the final products.

## Figures and Tables

**Table 1:** Some encapsulated food bioactive compounds.

Bioactive Compound	Basic Structure	Reference
Curcumin	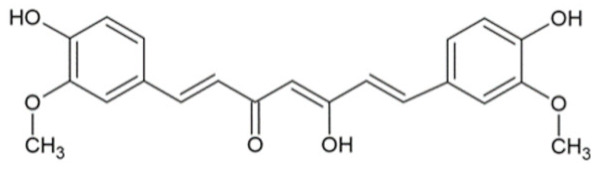	[28,29]
β-carotene	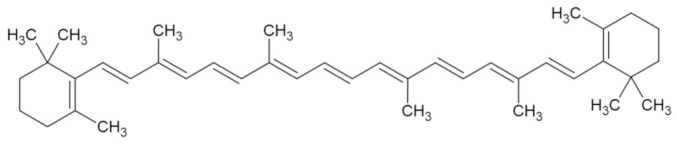	[30]
Astaxanthin	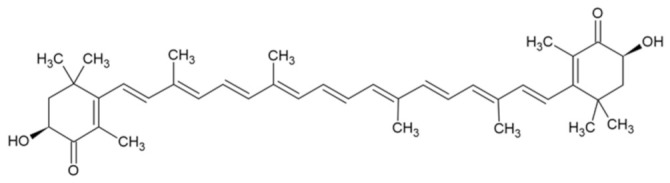	[31]
Fucoxanthin	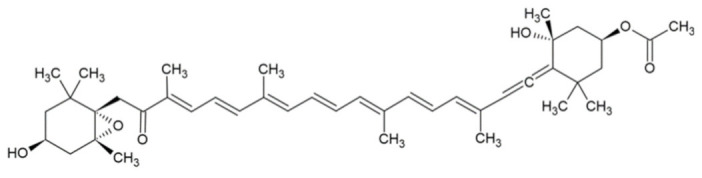	[32]
Theophylline	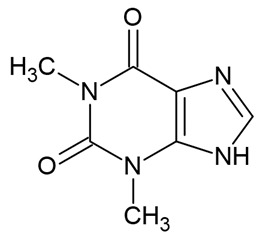	[33]
Caffeine	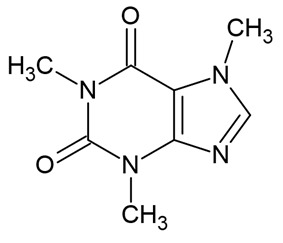	[34,35]
Vitamin B2	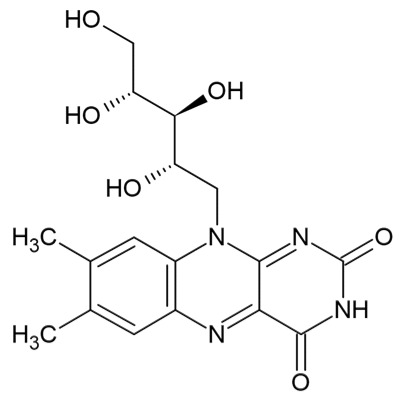	[36]
Vitamin A	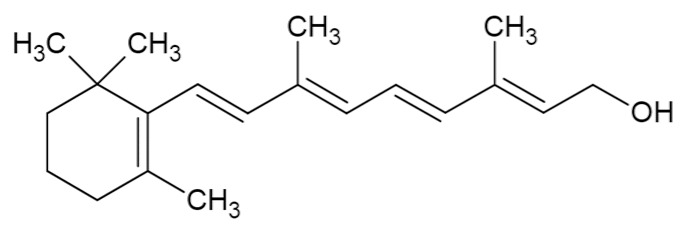	[37]
Linalool	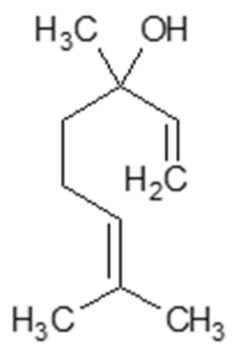	[38,39]
Coenzyme-Q10	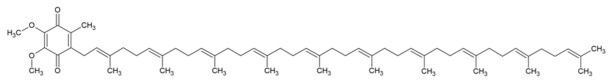	[40,41]
Omega-3 PUFAs^1^	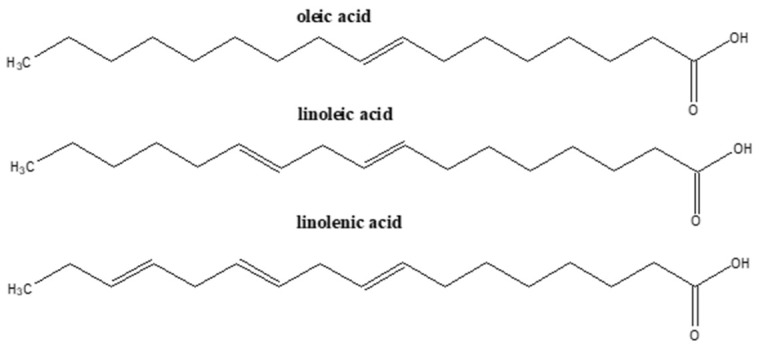	[31,42,43]
Eugenol	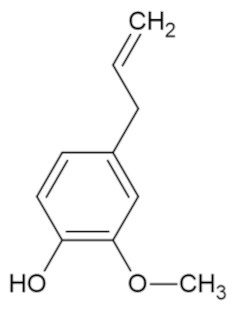	[44]
Thymol	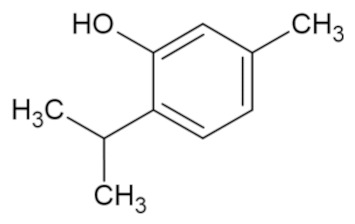	[44]

^1^ Polyunsaturated fatty acids.

**Table 2:** Technologies based on supercritical carbon dioxide for the encapsulation of bioactive compounds.

Technology	Bioactive Compound	Carrier Material	Process Conditions	Encapsulation Efficiency	Reference
PGSS ^1^	Theophylline	Hydrogenated palm oil	Pressure: 12–18 MPa Temperature: 60 °C	0.5–3.5%	[33]
PGSS ^1^	Caffeine	Glyceryl monostearate	Pressure: 13 MPa Temperature: 62 °C Time: 1 h	140 mg/g	[34]
PGSS ^1^	No bioactive	Rapeseed 70	Pressure: 7–18 MPa Temperature: 60–100 °C	- ^9^	[56]
PGSS ^1^	Anthocyanin concentrates from grape residues	Starch and silica	Pressure: 10–18 MPa Temperature: 25 °C	- ^9^	[57]
PGSS ^1^	Caffeine, Glutathione, Ketoprofen, silanized TiO2	Glyceryl monostearate, Hydrogenated castor oil	Pressure: 13 MPa Temperature: 72 °C Time: 1 h	- ^9^	[35]
PGSS ^1^	Lavandin essential oil	Polyethylene glycol	Pressure: 5.4–8.5 MPa Temperature: 76–84 °C	14–66%	[38]
PGSS ^1^	*Cydia pomonella* granulovirus	Palm oil-based fat and lecithin-based surfactant	Pressure: 10 MPa Temperature: 65 °C	- ^9^	[58]
PGSS ^1^	Co-enzyme Q10	Polyethylene glycol	Pressure: 10–25 MPa Temperature: 75–80 °C Time: 30 min	- ^9^	[40]
PGSS ^1^	Garlic essential oil	Polyethylene glycol	Pressure: 15.76–20.34 MPa Temperature: 50–62 °C	26.10–48.93%	[59]
PGSS ^1^	Mackerel lecithin	Polyethylene glycol	Pressure: 15–30 MPa Temperature: 40–50 °C Time: 1 h Stirring speed: 250 rpm	- ^9^	[60]
PGSS ^1^	β-carotene	Poly-(ε-caprolactone)	Pressure: 11 and 15 MPa Temperature: 50–70 °C Time: 240 min	306–336 ppm	[30]
PGSS ^1^	Linalool and lavandin essential oil	Poly-(ε-caprolactone)	Pressure: 6–11 MPa Temperature: 50–70 °C Time: 2 h	11–50% for linalool 13–45% for lavandin oil	[39]
PGSS ^1^	Squid lecithin	Polyethylene glycol	Pressure: 20–30 MPa Temperature: 40–50 °C Stirring speed: 200–400 rpm Time: 1 h	- ^9^	[61]
PGSS ^1^	Hydroxytyrosol-rich concentrate	Glycerol monostearate	Pressure: 13 MPa Temperature: 62 °C Time: 30 min	- ^9^	[62]
PGSS ^1^	Curcuminoids extract	Polyethylene glycol	Pressure: 16 MPa Temperature: 50 °C Time: 120 min	- ^9^	[63]
PGSS ^1^	Wheat germ oil	Polyethylene glycol	Pressure: 10–30 MPa Temperature: 40–50 °C Time: 1 h	- ^9^	[64]
CO_2_-expanded lipid mixture	Peppermint essential oil	Fully hydrogenated soybean oil	Pressure: 20 MPa Temperature: 57 °C Stirring speed: 1000 rpm	39–47.5%	[65]
PGSS ^1^	Mackerel reaction oil	Polyethylene glycol	Pressure: 10, 15, 20 MPa Temperature: 45–55 °C	- ^99^	[66]
PGSS ^1^	Coffee oil flavor	Polyethylene glycol	Pressure: 20–30 MPa Temperature: 40–50 °C Stirring speed: 300 rpm Time: 1 h	79.78%	[67]
PGSS ^1^	Anthocyanins from Elderberry (*Sambucus nigra*)	Palm fat	Pressure: 10 MPa Temperature: 60 °C Time: 2 h	- ^9^	[68]
PGSS ^1^	Spearmint essential oil	Fully hydrogenated canola oil	Pressure: 12.2 MPa Temperature: 60 °C Stirring speed: 20 Hz Time: 1 h	96%	[69]
PGSS ^1^	Menthol	Beeswax	Pressure: 6–20 MPa Temperature: 60 °C	60%	[70]
Modified PGSS	Vitamin B2	Fully Hydrogenated canola oil	Pressure: 10–25 MPa Temperature: 65 °C Time: 1 h	12–48%	[36]
PGSS ^1^	Citrus oil	Polyethylene glycol	Pressure: 20–40 MPa Temperature: 40–50 °C Stirring speed: 400 rpm Time: 1 h	43.95–83.87%	[71]
PGSS ^1^	Omega-3 PUFAs and astaxanthin-rich salmon oil	Polyethylene glycol	Pressure: 15–25 MPa Temperature: 45–55 °C Time: 1 h	62.19–79.20%	[31]
PGSS ^1^	Fucoxanthin-rich oil	Polyethylene glycol	Pressure: 10–30 MPa Temperature: 45–65 °C Stirring speed: 400 rpm Time: 1 h	62.41–81.85%	[32]
PGSS ^1^	Eucalyptol	Polyethylene glycol Polycaprolactone	Pressure: 8 MPa Temperature: 45 °C Stirring speed: 150 rpm Time: 1 h	60.69–77.36%	[72]
PGSS ^1^	Limonene	Modified starches	Pressure: 10–12 MPa Temperature: 50–60 °C Stirring speed: 1250 rpm Time: 45 min	86%	[73]
PGSS ^1^	Nimodipine Fenofibrate o-vanillin	Polyethylene glycol Polyoxyethylene stearyl ether	Pressure: 10–25 MPa Temperature: 45–60 °C Time: 1 h	Nimodipine: 59.7–98.82 Fenofibrate: 67–93.67% o-vanillin: 68.78–99.31	[74]
PGSS ^1^	Brewer’s spent grain oil	Polyethylene glycol	Pressure: 10–20–30–35 MPa Temperature: 45–55 °C Time: 1 h	73.5%	[75]
PGSS-drying	Green tea extracts	- ^9^	Pressure: 5.9–10 MPa Pre-expansion temperature: 130 °C	- ^9^	[76]
PGSS-drying	Lavandin oil	Modified OSA-starch from waxy maize	Pressure: 9–12.4 MPa Pre-expansion temperature: 100–131 °C Spray tower temperature: 60–75 °C	6–55%	[38]
PGSS-drying	Lavandin essential oil	Soybean lecithin	Pressure: 6–10.3 MPa Pre-expansion temperature: 103–128 °C Spray tower temperature: 38–52 °C	6–14.5%	[77]
PGSS-drying	β-carotene	Soybean lecithin	Pressure: 8.1–10.3 MPa Pre-expansion temperature: 102–132 °C Spray tower temperature: 55 °C Time: 60 min	29–58%	[78]
PGSS-drying	Fish oil	Chitosan, Maltodextrin	Pressure: 11–25.7 MPa Spray tower temperature: 64–119 °C	- ^9^	[79]
PGSS-drying	Resveratrol	β-glucans Soy-bean lecithin	Pressure: 9.5 MPa Pre-expansion temperature: 125 °C Spray tower temperature: 65–70 °C	- ^9^	[80]
PGSS-drying	Epigallocatechin gallate	Octenyl-succinic-anhydride modified starch Soybean lecithin β-glucan (Glucagel™)	Pressure: 9.5 MPa Pre-expansion temperature: 124.85 °C Spray tower temperature: 69.85 °C	OSA-starch: 80.5% Lecithin: 75.8% β-glucan: 77.4%	[81]
PGSS-drying	Quercetin	Poly-(ethylene glycol)-block-poly-(propylene glycol)-block- poly-(ethylene glycol) Soy-bean lecithin	Pressure: 7.68–11.77 MPa Pre-expansion temperature: 109.4–132.5 °C Spray tower temperature: 64.7–75.1 °C	- ^9^	[82]
PGSS-drying	Omega-3	Octenyl-succinic-anhydride modified starch	Pressure: 10 MPa Pre-expansion temperature: 110 °C Spray tower temperature: 55 °C	97.9%	[83]
PGSS-drying	Rice bran oil	Pea protein isolate (PPI) and Maltodextrin (MD)	Pressure: 10 MPa Pre-expansion temperature: 105 °C Spray tower temperature: 55 °C	53%	[84]
PGX ^2^	Co-enzyme Q10	β-glucan	Pressure: 10–30 MPa Temperature: 32–50 °C	- ^9^	[85,86]
RESS ^3^	Glass beads	Stearyl alcohol (1-Octadecanol)	Pressure: 8 MPa Temperature: 55 °C	- ^9^	[87]
RESS ^3^	Anthocyanin extract obtained from jabuticaba (*Myrciaria cauliflora*) skins	Polyethylene glycol	Pressure: 10–35 MPa Temperature: 40–50 °C Time: 30 min	79.78%	[88]
RESS ^3^	Rose essential oil	Phosphatidylcholine and cholesterol	Pressure: 20–30 MPa Temperature: 60–70 °C Time: 2 h	73.16–90.28%	[89]
RESS ^3^	Essential oil of *Atractylodes macrocephala* Koidz	Phosphatidylcholine and cholesterol	Pressure: 15–30 MPa Temperature: 65 °C Time: 1 h	82.18%	[90]
RESS ^3^	*Curcuma Longa* L. extracts	- ^9^	Pressure: 8–35 MPa Temperature: 50 °C Time: 10–30 min	- ^9^	[91]
RESS ^3^	Rutin and anthocyanin-rich extract	Polyethylene glycol	Pressure: 20 MPa Temperature: 40 °C Time: 30 min	44.2%	[92]
RESS-N	Lysozyme and lipase	Polyethylene glycol Poly(methyl methacrylate) Poly(L-lactic acid) Poly(DL-lactide-co-glycolide)	Pressure: 20 MPa Temperature: 35 °C	- ^9^	[93]
SAS ^4^	Bixin (annatto seed extract)	Polyethylene glycol	Pressure: 10 MPa Temperature: 40 °C	62%	[92]
SAS ^4^	Lutein	Polylactic acid	Pressure: 8–10 MPa Temperature: 35–45 °C	90%	[94]
SAS ^4^	Lutein	Hydroxypropylmethyl cellulose phthalate	Pressure: 11–15 MPa Temperature:40–50 °C	88.41%	[95]
SAS ^4^	Lutein β-carotene	Polyethylene glycol	Pressure: 8–10 MPa Temperature: 15 °C	- ^9^	[96]
SAS ^4^	Lutein	Hydrogenated phosphatidylcholine	Pressure: 8–16 MPa Temperature: 35–55 °C	>90%	[97]
SAS ^4^	Vitamin D3	Hydrogenated phosphatidylcholine	Pressure: 8–12 MPa Temperature: 35–55 °C	98%	[98]
SAS ^4^	Polyphenols (green tea extract)	Poly(epsilon-caprolactone)	Pressure: 8–12 MPa Temperature: 11–34 °C	- ^9^	[99]
SAS ^4^	Rosemary antioxidants	Pluronic^®^ F 88Pluronic^®^ F 127	Pressure: 8–10 MPa Temperature: 25–50 °C	100%	[53]
SAS ^4^	Astaxanthin (Shrimp extract)	Pluronic^®^ F 127	Pressure: 10–12 MPa Temperature: 35–40 °C	74%	[100]
SAS ^4^	Quercetin	Pluronic^®^ F 127	Pressure: 10 MPa Temperature: 40 °C	35–56%	[101]
SAS ^4^	Passion fruit seeds oil	Poly(lactic-co-glycolic) acid	Pressure: 9–11 MPa Temperatures: 35 and 45 °C	67.8–91%	[102]
GAS ^5^	Rosemary extract	Polycaprolactone	Pressure: 20–30 MPa Temperature: 40 °C	82.8%	[54]
Supercritical impregnation	Lycopene	Hydrolysed collagen	Pressure: 15–25 MPa Temperature: 50–60 °C	84–94%	[103]
Supercritical impregnation	Lavandin essential oil	Modified OSA starch derived from waxy maize	Pressure: 10–12 MPa Temperature: 40–50 °C Time: 2 h	Lavandin oil: 22% Linalool: 22% Linalyl acetate: 51%	[104]
SEDS ^6^	Lutein	Zein	Pressure: 10–15 MPa Temperature: 32–45 °C	34.44–83.15%	[55]
SEDS ^6^	β-Carotene	Poly(hydroxybutirate-co-hydroxyvalerate)	Pressure: 8 MPa Temperature: 40 °C	7.75–55.54%	[105]
SEDS ^6^	β-Carotene	Poly(hydroxybutirate-co-hydroxyvalerate)	Pressure: 8–12 MPa Temperature: 30–70 °C	80%	[106]
SEDS ^6^	Grape seed extract	Poly(hydroxybutirate-co-hydroxyvalerate)	Pressure: 8–12 MPa Temperature: 35–45 °C	66.01%	[107]
SEDS ^6^	Pink pepper extract (PPE)	Poly(hydroxybutirate-co-hydroxyvalerate)	Pressure: 8–12.5 MPa Temperature: 35–55 °C	20.2–95.1%	[108]
SEDS ^6^	Astaxanthin	Poly(hydroxybutirate-co-hydroxyvalerate)	Pressure: 8–10 MPa Temperature: 35 °C	20.93–48.25%	[109]
SEDS ^6^	Puerarin	Poly(_L_-lactide)	Pressure: 12 MPa Temperature: 33 °C	39.4%	[110]
SFEE ^7^	Lysozyme	Poly(lactic-co-glycolic) Calcium carbonate	Pressure: 8–10 MPa Temperature: 33–40 °C Mixing speed: 1500 rpm Time: 25 min	60%	[111]
SFEE ^7^	Astaxanthin (Shrimp extract)	Pluronic^®^ F 127	Pressure: 10 MPa Temperature: 40 °C	93%	[100]
SFEE ^7^	Oleoresin of *Capsicum frutescens* pepper	Hi-Cap 100 modified starch	Pressure: 9–11 MPa Temperature: 40 °C	100%	[112]
SFEE ^7^	Low viscosity omega-3 rich fish oil	Polycaprolactone	Pressure: 8 MPa Temperature: 40 °C	12–43%	[113]
SFEE ^7^	β-caroteneLycopene	Octenyl succinyl modified starch	Pressure: 9–13 MPa Temperature: 80 °C	34–89%	[114]
SFEE ^7^	Quercetin	Poly-(ethylene glycol)-block-poly-(propylene glycol)-block- poly-(ethylene glycol)Soy-bean lecithin	Pressure: 7.91–10.48 MPa Temperature: 34.6–40.3 °C Time: 75–104 min Mixing speed: 1500 rpm	80.1–98.5%	[82]
SuperLip ^8^	Phospholipids	Soy lecithin	Pressure: 30 MPa Temperature: 40–50 °C Time: 60 min	-^9^	[115]
SuperLip ^8^	Lutein	Soy lecithin	Pressure: 3–30 MPa Temperature: 40–65 °C Mixing speed: 550 rpm Time: 60 min	56.7–97.0%	[46]
SuperLip ^8^	Anthocyanin	Soy lecithin	Pressure: 30 MPa Temperature: 50 °C Mixing speed: 550 rpm Time: 60 min	50.6%	[116]
SuperLip ^8^	BSA	Soybean phosphatidylcholine and phosphatidyl glycerol	Pressure: 12.5–17.5 MPa Temperature: 40–70 °C Mixing speed: 400 rpm Time: 30 min	92–98%	[117]
SuperLip ^8^	Eugenol α-lipoic acid	Lipids and lipophilic compounds	Pressure: 10 MPa Temperature: 35–40 °C	68.1–94.2%	[118]

^1^ Particles from Gas Saturated Solution; ^2^ Pressurized Gas Expanded Technology; ^3^ Rapid Expansion of Supercritical Solutions; ^4^ Supercritical Anti-Solvent Process; ^5^ Gas Anti-Solvent; ^6^ Solution Enhanced Dispersion by Supercritical Fluid Process; ^7^ Supercritical Fluid Extraction of Em50ulsions; ^8^ Supercritical Liposomes; ^9^ Data not available.
